# The causal relationship between breast cancer and frozen shoulder: A two-sample Mendelian randomization

**DOI:** 10.1097/MD.0000000000035630

**Published:** 2023-11-03

**Authors:** Guang-Hua Deng, Yong-Kang Wei

**Affiliations:** a Ya’an Hospital of Traditional Chinese Medicine, Ya'an, Sichuan, China; b The Fourth Clinical College of Xinjiang Medical University, Urumqi, Xinjiang, China.

**Keywords:** breast cancer, frozen shoulder, Mendelian randomization

## Abstract

To investigate the causal relationship between breast cancer and frozen shoulder using a Mendelian randomization (MR) approach. Pooled data from a large-scale genome-wide association study were used. Genetic loci that were independent of each other and associated with breast cancer and frozen shoulder in populations of European ancestry were selected as instrumental variables. Inverse variance weighting was used as the primary analysis method. Weighted median (WME) and MR-Egger were used as complementary analysis methods to assess causal effects. To explore the causal relationship between breast cancer and frozen shoulder. Sensitivity test analysis was performed using heterogeneity test, multiple validity test, and leave-one-out analysis to explore the robustness of the results. Inverse variance weighting results showed an OR (95% CI) of 1.02 (1.00–1.04), *P* = .048, indicating that breast cancer is a risk factor for a frozen shoulder. And the test revealed no heterogeneity and pleiotropy, and the sensitivity analysis also showed robust results. In this study, genetic data were analyzed and explored using two-sample MR analysis, and the results showed that the incidence of frozen shoulder was higher in breast cancer patients, suggesting that screening for frozen shoulder in breast cancer patients should be increased.

## 1. Introduction

Frozen shoulder, also known as adhesive capsulitis,^[1]^ is a pathological condition characterized by pain and limited joint motion in the shoulder.^[2]^ There are usually no significant findings in the patient’s history, clinical examination, or imaging evaluation to explain the loss of motion or pain.^[3,4]^ Breast cancer is the most common cancer in the female population.^[5,6]^ Breast cancer is most commonly treated surgically,^[7,8]^ and mastectomy may negatively affect the size and movement of shoulder muscles, and patients treated with postoperative radiation therapy frequently develop arm and shoulder disorders.^[9]^ In recent years, several studies have investigated the relationship between breast cancer and adhesive capsulitis, but the findings have been mixed.^[9,10]^

In traditional epidemiological research methods, the causal inferences obtained are considered to be of limited value because of the influence of confounding factors and reverse causality.^[11]^ In contrast, Mendelian randomization (MR), a genetic epidemiological method, is a useful tool to assess the causal role of breast cancer and frozen shoulder.^[12]^ By using genetic variants such as single nucleotide polymorphism (SNP) as instrumental variables that can modify disease risk factors or exposures, MR studies can strengthen causal inferences about exposure-outcome associations.^[13]^ According to Mendel law of inheritance, genetic variants are not susceptible to confounding factors because they are randomly assigned during gamete formation.^[14]^ In addition, confounding factors and reverse causality correlations can be minimized because genotypes cannot change with disease progression.^[15]^

To this end, we conducted a two-sample MR study to examine the association of genetic susceptibility to breast cancer with frozen shoulder risk factors. We aimed to provide important evidence for the causal role of breast cancer in causing a frozen shoulder.

## 2. Data and methods

### 2.1. Data sources

The largest sample size of genome-wide association study (GWAS) data for breast cancer and frozen shoulder was obtained through the IEU OpenGWAS project (mr cieu. ac. UK). The website was accessed on 2023-06-06. The final population source for all data was Europe, of either sex. Including breast cancer (ieu-a-1162) containing 195,514 SNPs, 46,785 in the observation group and 42,892 in the control group; frozen shoulder (ebi-a-GCST90000512) containing 15,184,371 SNPs with a sample size of 451,099.This study was a reanalysis of previously collected and published data and therefore did not require additional ethical approval.

### 2.2. Conditioning of SNP as an instrumental variable

First, the instrumental variables were highly correlated with exposure, with *F* > 10 as a strong correlation criterion.^[16]^ Secondly, the instrumental variable was not directly correlated with the outcome, but only influenced the outcome through exposure, meaning that there was no genetic pleiotropy. In this study, the MR-Egger regression model with a nonzero intercept term (*P* < .05) indicated the absence of genetic pleiotropy.^[17]^ Third, instrumental variables were not related to unmeasured confounding.^[18]^ Finally, the human genotype–phenotype association database Phenoscanner V2 was searched for phenotypes associated with instrumental variables at genome-wide significance levels to determine whether these SNPs were associated with potential risk factors.^[19]^

### 2.3. SNP screening

Significant SNPs were screened from the GWAS summary data of breast cancer (*P* < 5 × 10^−8^ was used as the screening condition)^[20]^; the linkage disequilibrium coefficient *r*^2^ was set to 0.001 and the width of the linkage disequilibrium region was 10,000 kb to ensure that each SNP was independent of each other.^[21]^ The above-screened breast cancer-related SNPs were extracted from the GWAS summary data of frozen shoulder, while SNPs directly related to the outcome index were excluded (*P* < .05). The *F*-value of each SNP was calculated, and SNPs with weak instrumental variables (*F*-value < 10) were excluded.^[22]^ And the human genotype–phenotype association database was queried to screen for potentially associated risk factor SNPs and to exclude them.^[23]^

### 2.4. Causality validation methods

The causal relationship between exposure (breast cancer) and outcome (frozen shoulder) was verified mainly using inverse variance weighting (IVW), supplemented by weighted median (WME) and MR-Egger MR analysis methods, using SNPs as instrumental variables.

### 2.5. Sensitivity analysis

Various methods were used for sensitivity analysis. First, the Cochran Q test was used to assess the heterogeneity among the individual SNP estimates, and a statistically significant Cochran Q test proved that the analysis was significantly heterogeneous. Second, the MR pleiotropy residual sum and outlier (MRPRESSO) was used to validate the results in the IVW model, correct for the effects of outliers, and if outliers existed, they were excluded and the analysis was repeated. Third, the MR-Egger intercept test was used to test the horizontal multiplicity of SNPs. If the intercept term in the MR-Egger intercept test analysis was statistically significant, it indicated that the MR analysis had significant horizontal multiplicity. Fourth, leave-one-out analyses were performed by removing a single SNP at a time to assess whether the variation drove the association between the exposure and outcome variables. Fifth, funnel plots and forest plots were constructed to visualize the results of sensitivity analyses. *P* < .05 suggests a potential causal relationship for MR analysis and is statistically significant. All statistical analyses were performed using the “TwoSampleMR” package in R software version 4.3.0.

## 3. Results

### 3.1. Instrumental variables

The current study screened 53 SNPs that were strongly associated with breast cancer (*P* < 5 × 10^−8^) without chain imbalance (*r*^2^ < 0.001, kb = 10,000). Fifty-three SNPs remained by taking intersection with SNPs in the GWAS pooled data from frozen shoulder and also excluding SNPs directly associated with outcome indicators. In our study, each SNP had an *F* value >10, indicating no weak instrumental variables (Table 1). We searched the human genotype–phenotype association database and no potentially relevant risk factor SNPs were found.

**Table 1:** Information on the final screening of breast cancer SNPs from GWAS data (n = 53).

ID	SNP	Effect_Allele	Other_Allele	β	SE	*P*	*F*
1	rs10816625	G	A	0.1175	0.0198	2.96E−09	35
2	rs10941679	G	A	0.124	0.0111	3.57E−29	124
3	rs10995194	C	G	−0.154	0.0137	2.23E−29	126
4	rs11049407	G	A	−0.058	0.0105	3.60E−08	30
5	rs11205277	G	A	0.0569	0.0098	5.59E−09	33
6	rs11242675	T	C	−0.0554	0.0101	4.29E−08	30
7	rs11249433	G	A	0.0874	0.0099	7.66E−19	77
8	rs11780156	T	C	0.0722	0.013	3.06E−08	30
9	rs11814448	C	A	0.229	0.033	3.99E−12	48
10	rs12575663	A	G	−0.0581	0.0097	2.31E−09	35
11	rs12653202	C	A	0.1871	0.013	1.00E−200	207
12	rs1292011	G	A	−0.0828	0.0099	6.19E−17	69
13	rs13066793	G	A	−0.1061	0.017	3.98E−10	38
14	rs13113	A	T	−0.7467	0.099	4.61E−14	56
15	rs13329835	G	A	0.0775	0.0115	1.48E−11	45
16	rs1432679	T	C	0.068	0.0098	3.28E−12	48
17	rs16854041	G	A	−1.3818	0.1498	2.92E−20	85
18	rs16857609	T	C	0.0749	0.0109	7.23E−12	47
19	rs17136641	A	G	0.1961	0.0136	1.00E−200	207
20	rs17271951	C	T	0.2221	0.0109	1.00E−200	415
21	rs17356907	G	A	-0.094	0.0107	1.20E−18	77
22	rs17465052	A	G	−0.1104	0.0099	7.55E−29	124
23	rs17817449	G	T	−0.0702	0.0099	1.41E−12	50
24	rs1875622	T	C	−0.056	0.01	2.27E−08	31
25	rs2236007	A	G	−0.0766	0.012	1.69E−10	40
26	rs2628316	G	A	−0.0828	0.0108	1.57E−14	58
27	rs2736108	T	C	−0.0623	0.0107	6.42E−09	33
28	rs2747652	C	T	−0.0684	0.0098	2.59E−12	48
29	rs2896680	T	A	−0.0807	0.0112	5.15E−13	51
30	rs3760982	G	A	0.0549	0.0097	1.68E−08	32
31	rs4237533	G	A	−0.1674	0.0098	1.00E−200	291
32	rs4442975	T	G	−0.1374	0.0097	1.00E−200	200
33	rs4808801	G	A	−0.0741	0.0102	4.70E−13	52
34	rs4849887	C	T	−0.0957	0.0166	8.23E−09	33
35	rs571978	G	A	−0.1029	0.0097	4.24E−26	112
36	rs581235	C	T	−1.4017	0.1727	4.86E−16	65
37	rs6001930	C	T	0.1163	0.0153	3.44E−14	57
38	rs616488	G	A	−0.0579	0.0103	2.13E−08	31
39	rs620315	A	G	−0.0818	0.01	3.39E−16	66
40	rs6430298	A	C	0.0616	0.01	8.02E−10	37
41	rs6472903	T	G	−0.094	0.0129	3.07E−13	53
42	rs648354	A	G	−0.1172	0.0101	6.60E−31	134
43	rs6762644	G	A	0.0633	0.0099	1.83E−10	40
44	rs7077039	C	T	0.059	0.0097	1.23E−09	36
45	rs720475	A	G	−0.0625	0.0112	2.49E−08	31
46	rs7297051	T	C	−0.1241	0.0115	4.09E−27	116
47	rs739874	G	A	−0.0907	0.0111	3.27E−16	66
48	rs76472065	T	C	−0.0918	0.0142	9.48E−11	41
49	rs7697216	C	T	−0.1208	0.0153	2.96E−15	62
50	rs873823	A	G	0.0799	0.0099	6.89E−16	65
51	rs9397437	A	G	0.182	0.0189	7.36E−22	92
52	rs941764	G	A	0.0622	0.0102	1.02E−09	37
53	rs9693444	C	A	0.068	0.0103	4.60E−11	43

### 3.2. Causal relationship between breast cancer and frozen shoulder

**Figure 1.**
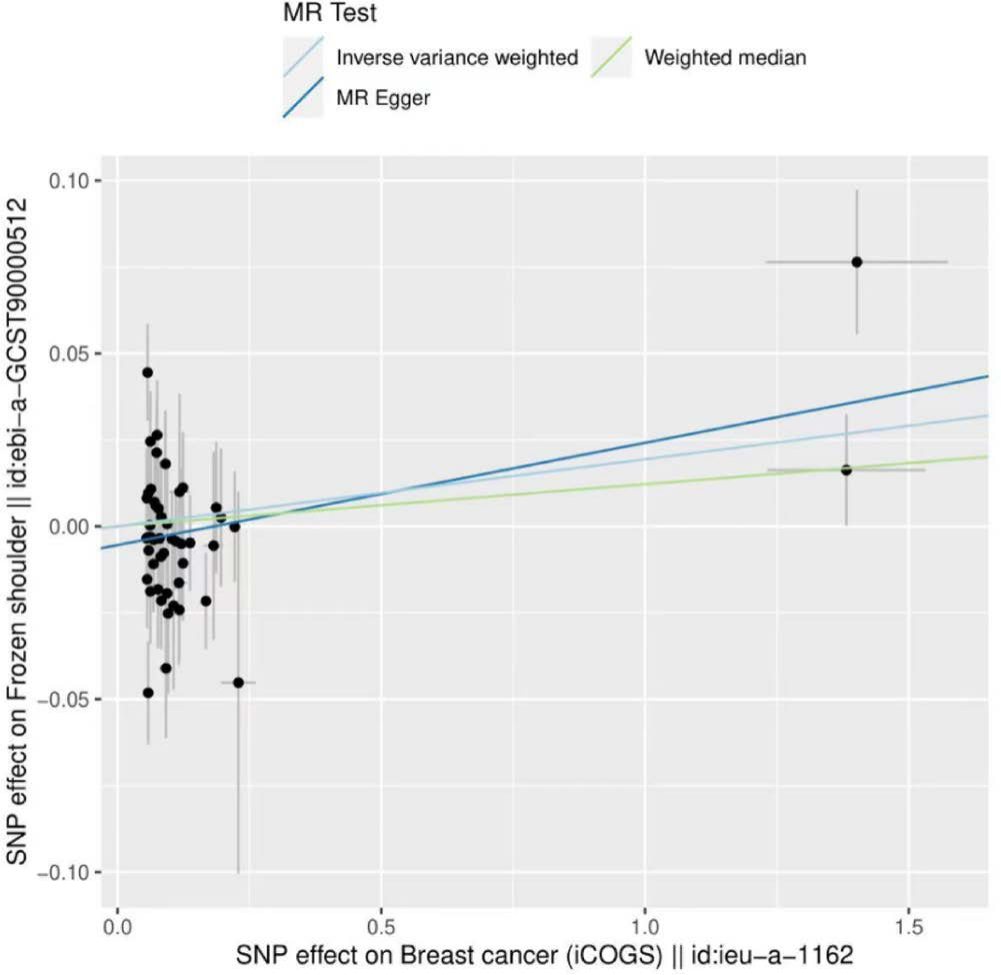
Scatter plot of breast cancer and frozen shoulder.

**Figure 2.**
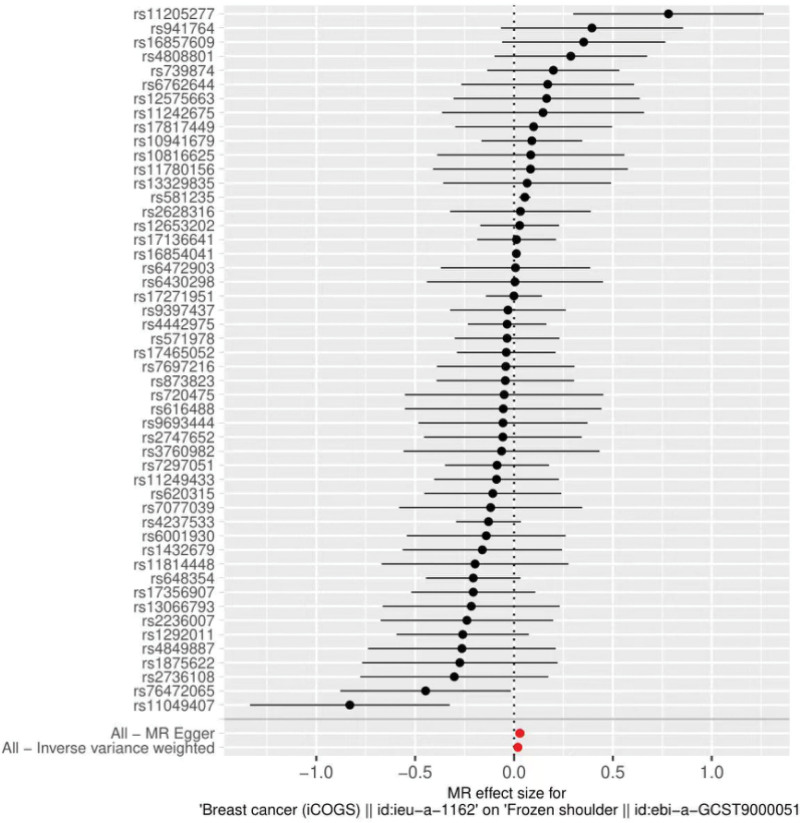
Forest plot of breast cancer and frozen shoulder.

The results of both IVW and MR Egger showed a causal relationship between breast cancer and frozen shoulder. IVW: OR = 1.02, 95% CI = 1.00–1.04, *P* = .048; MR Egger: OR = 1.03, 95% CI = 1.01–1.05, *P* = .010 (Table 2). We can see from both the scatter plot (Fig. 1) and the forest plot (Fig. 2) that breast cancer increases the risk of developing frozen shoulder.

**Table 2:** MR regression results of the 3 methods.

Method	β	SE	OR (95%CI)	*P*
IVW	0.019	0.010	1.02 (1.00–1.04)	.048
WME	0.012	0.012	1.01 (0.99–1.04)	.327
MR-Egger	0.029	0.110	1.03 (1.01–1.05)	.010

### 3.3. Sensitivity analysis

**Figure 3.**
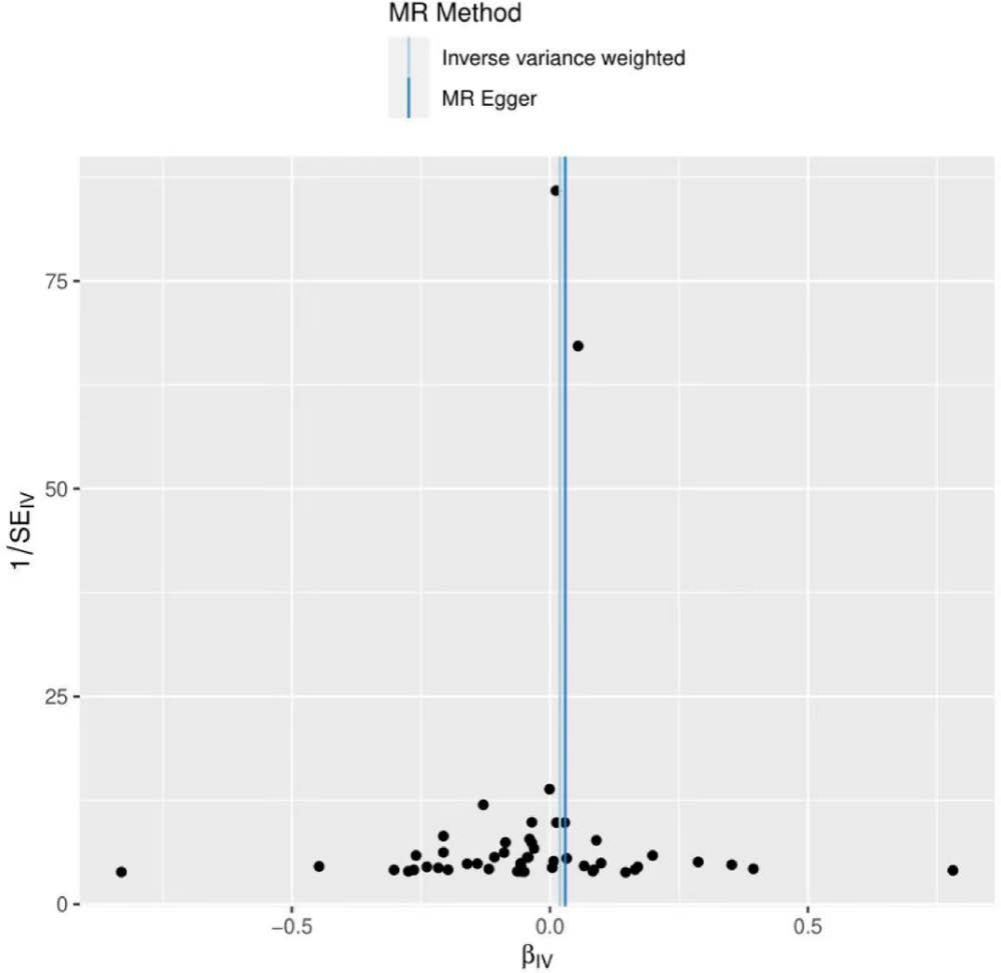
Funnel plot of breast cancer and frozen shoulder.

**Figure 4.**
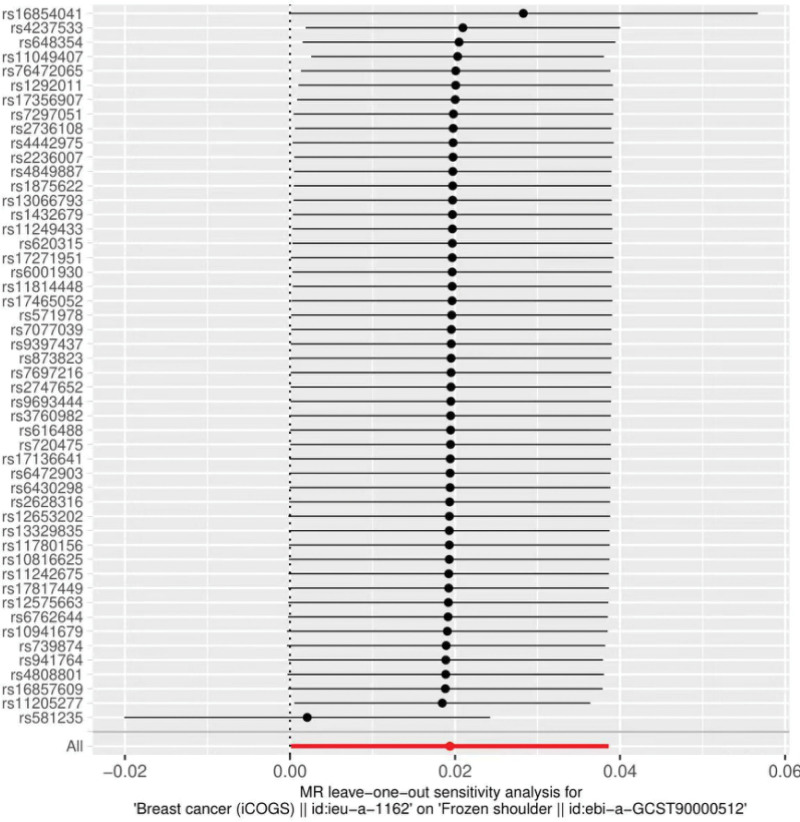
Analysis of breast cancer and frozen shoulder by the leave-one-out method.

The heterogeneity test (Cochran Q test, *P* = .067) was performed using the IVW method and the results suggested that there was no heterogeneity. A funnel plot was drawn to show the heterogeneity results, as shown in Figure 3. MR-PRESSO was used to screen for SNPs that might cause heterogeneity, and no SNPs were found to cause heterogeneity in the results. The results of the global test by MR-PRESSO suggested that there was no pleiotropy (*P* = .066). The IVW method was used by default for the leave-one-out method, and as seen in Figure 4, the results of the remaining SNPs after eliminating any of them were on the right side of the valid line, indicating that the results were robust.

## 4. Discussion

It is known that breast cancer may be an observable risk factor for a frozen shoulder, but the causality of this association is unclear. Our MR study aimed to reveal the causal relationship between breast cancer and a frozen shoulder. The results by two-sample MR showed that breast cancer was a risk factor for a frozen shoulder with an OR (95% CI) of 1.02 (1.00–1.04), indicating that the risk of the frozen shoulder was 1.02 times higher in breast cancer patients compared to the general population.

In a cross-sectional study,^[24]^ Ch et al retrospectively analyzed 785 patients and found that the incidence of frozen shoulder occurred in 3.8% of patients after breast cancer surgery, which is significantly higher than the general population. Another study^[9]^ followed up 217 female patients who underwent breast cancer surgery 13 to 18 months after surgery and found a cumulative incidence of the frozen shoulder of 10.3%, which was also significantly higher than the general population. A study by Pederse et al^[25]^ found that the risk of developing a frozen shoulder after breast cancer surgery was higher than in the population without breast cancer (OR: 1.51, 95% CI: 1.02–2.15).

However, Louis et al^[10]^ found a 10-year incidence of the frozen shoulder of 3.6% (*P* = .317) in both the breast cancer and no breast cancer groups in a retrospective study of 52,524 women. cox regression analysis further showed no significant association between breast cancer and frozen shoulder (OR = 0.96, 95% CI = 0.86–1.08).

Different conclusions emerged from previous studies, probably because retrospective studies are susceptible to confounding factors and reverse causality, and therefore the causal inferences obtained are considered to be of limited value. In contrast, MR analysis is a new epidemiological approach that uses genetic variation as an instrumental variable of exposure to enhance causal inferences. This approach reduces the effects caused by confounding factors.^[26]^

The present study confirms the risk factors for breast cancer and frozen shoulder from a genetic perspective. Although it is not clear how breast cancer plays this role, previous studies have suggested possible potential mechanisms. Since the majority of breast cancer patients are treated with aromatase inhibitors. Although aromatase inhibitor therapy may reduce the risk of disease recurrence, it may also increase the risk of a frozen shoulder.^[9]^

The results of the present study are consistent with the findings of most previous studies that breast cancer is a risk factor for the development of a frozen shoulder. People with breast cancer are more likely to develop frozen shoulders. Therefore, screening for frozen shoulder should be increased for the breast cancer population, early detection of patients with frozen shoulder and timely treatment will be beneficial to the prognosis of patients.

At the same time, there are some limitations to this study. First, because all data were from a population of European ancestry, the results do not represent a truly randomized population sample and do not apply to other so races. Second, although various sensitivity analyses have been performed in this study to test the hypotheses of the MR study, it is also difficult to completely rule out horizontal pleiotropy of instrumental variables. Finally, the current sample size of GWAS data is still not large enough, and more in-depth studies using more GWAS data are needed in the future.

## 5. Conclusion

In conclusion, this study used a two-sample MR analysis to analyze and explore the genetic data, and the results showed a higher incidence of frozen shoulder in breast cancer patients, suggesting that screening for frozen shoulder in breast cancer patients should be increased.

## Author contributions

**Conceptualization:** Yong-Kang Wei.

**Data curation:** Guanghua Deng.

**Formal analysis:** Guanghua Deng.

**Investigation:** Guanghua Deng.

**Methodology:** Guanghua Deng.

**Project administration:** Guanghua Deng.

**Resources:** Guanghua Deng.

**Software:** Guanghua Deng.

**Supervision:** Guanghua Deng.

**Validation:** Guanghua Deng.

**Visualization:** Guanghua Deng.

**Writing – original draft:** Guanghua Deng.

**Writing – review & editing:** Yong-Kang Wei.
